# Case Report: Kidney Transplantation in a Patient With Acquired Agammaglobulinemia and SLE. Issues and Challenges

**DOI:** 10.3389/fmed.2021.665475

**Published:** 2021-03-12

**Authors:** Paraskevi Pavlakou, Marios Papasotiriou, Theodoros Ntrinias, Alexandra Kourakli, Adamantia Bratsiakou, Dimitrios S. Goumenos, Evangelos Papachristou

**Affiliations:** ^1^Department of Nephrology and Renal Transplantation, University Hospital of Patras, Achaia, Greece; ^2^Hematology Division, Department of Internal Medicine, University Hospital of Patras, Achaia, Greece

**Keywords:** kidney transplantation, immunodeficiency, systemic lupus erythematosus, hypogammaglobulinemia, intravenous immunoglobulin

## Abstract

Lupus nephritis in the context of Systemic Lupus Erythematosus (SLE) is characterized by an unpredicted course with remissions and flare-ups. Among others, it remains a significant cause of end-stage kidney disease (ESKD) in relatively young patients. Therapeutic regimens with newer immunosuppressive agents have been introduced in order to control SLE clinical manifestations more efficiently and limit organ damage induced by immune complex formation and sustained inflammation. Treatment is usually long-term, and the cumulative impact of immunosuppression is expressed through the increased frequency of infections and neoplasms. However, if the observed immunity dysregulation is secondary and pharmaceutically induced or there is a pre-existing, primary immunodeficiency that shares common pathogenetic pathways with SLE's autoimmunity is not always clear. Herein, we present the case of a 39-year-old woman, that reached ESKD due to lupus nephritis. After an upper respiratory cytomegalovirus (CMV) infection and concomitant CMV reactivations the investigation revealed significant immunodeficiency. Not long after the initiation of intravenous immunoglobulin (IVIG) administration, patient received a cadaveric kidney transplant. IVIG was continued along with standard immunosuppression so that both recurrent infections and allograft rejection are avoided. Patient is closely monitored, and her post-transplant course is remarkably satisfying so far. ESKD patients with immunodeficiency syndromes should not be excluded by definition from kidney transplantation.

## Introduction

### Systemic Lupus Erythematosus and Autoimmunity

Lupus nephritis remains a significant cause of end-stage kidney disease (ESKD). Despite the improvement in renal survival during the past decades due to new therapies, the 5-year incidence of ESKD in patients with lupus nephritis is 11% (1). Lupus clinical course is characterized by periods of remissions and unpredicted flareups that dictate the need and even the increase of immunosuppressive treatment in order to control symptoms. The cumulative impact of immunosuppression on patients is reflected on the incidence of infections (2) and even neoplasms (3) on the long-term, making clear the need for minimization of toxicity. Nevertheless, the dysregulations of immunity that are present are related to the pathophysiology of lupus with emphasis on coexisting genetic variations, lymphopenia and hypocomplementemia aggravating further the susceptibility to infections (4).

Systemic lupus erythematosus (SLE) is a systemic multi-organ chronic disease with genetic predisposition and environmental triggering that lead to the production of autoantibodies against nuclear antigens which are responsible for the disease manifestations. Shared genetic pathways determine the complex interplay between SLE and immunity disorders (4, 5). The underlying deficiency in complement components, the defective immunoglobulin synthesis (partial deficiencies in IgA and IgM mostly) and/or the co-existence of granulomatous or other autoimmunity disorders (i.e., Wiskott-Aldrich syndrome, autoimmune lymphoproliferative syndrome etc) are the main parameters of clinically expressed immunodeficiency and SLE (6). Under these circumstances, there is a constant activation of the immune system against self-antigens, a dysregulated immune complex formation and degradation that lead to unpredicted tissue damage enhancing further any pre-existing tendency toward autoimmunity and SLE as well (4, 5).

### Immunodeficiency Syndromes

The range of immunodeficiency syndromes (IS) is wide and consists of primary forms with genetic predisposition and secondarily induced immunity dysregulations that lead to hypogammaglobulinemia and frequent infections (7). Diagnosis demands broad investigation and exclusion of secondary causes. Despite efforts and consensus reports on diagnostic criteria for IS there are overlapping cases that do not fall into one category in specific, termed unspecified hypogammaglobulinemia (8) and mirror the complexity in diagnosing and classifying IS (8, 9). The exact prevalence of immunodeficiency syndromes is not known as the field is currently under study but secondary forms of IS are adding up making the interest for the establishment of a classification even greater (10). Secondary immunodeficiency has been described in patients suffering from hematologic malignancies (lymphoma, multiple myeloma) as well as those under immunosuppressants. A special group of patients are solid organ transplant patients who are likely to develop hypogammaglobulinemia post transplantation but especially those with pre-existing gamma globulin dysregulations and an intention to receive an allograft plus immunosuppressants. As these patients are highly prone to infections and the aetiologic treatment of secondary IS with immunosuppressants' withdrawal is not an option, close monitoring and proper translation of laboratory parameter results is a possible strategy. For example, low levels of complement components indicate an increased risk for bacterial infections while enzyme-linked immune absorbent spot (ELISPOT) and flow cytometry low response of anti-CD8 lymphocytes to cytomegalovirus (CMV) antigens reflect increased probability for CMV infection (11).

B-cell depleting therapies are established as a highly effective treatment option not only in lymphoproliferative disorders but also in collagen inflammatory diseases. However, one cannot avoid recognizing the induced rise in the rate of infections that complicate patients' course that may even be fatal (12), in the context of secondary IS.

The main axis of treatment in patients with IS is the management of infections and their prevention through prophylactic antibiotics and antivirals. The substitution of gamma globulins is another option that seems to benefit patients with significantly low levels of IgG (<4 g/L), recurrent infections with failure of prophylactic antibiotics and immunity disorders namely failure in post-exposure immunization while the route of administration (subcutaneous or intravenous) doesn't affect the effect of the regimen (13).

### Case Report

A 39-year-old female patient was referred to our Nephrology Department during late 2009. She was newly diagnosed with SLE after a spontaneous miscarriage during the second trimester of pregnancy. Due to significant proteinuria and microscopic hematuria a kidney biopsy was performed which revealed crescentic focal segmental glomerulonephritis [class III lupus nephritis with crescents according to WHO classification (14)]. She was initially managed with a combined therapeutic regimen of cyclophosphamide, corticosteroids and azathioprine which was modified due to poor response, to mycophenolic acid with the addition of rituximab. However, despite the absence of extra-renal manifestations of lupus, kidney function gradually deteriorated and patient reached ESKD about 4 years after the initiation of immunosuppression. Peritoneal dialysis was the type of renal replacement therapy that was initiated with the concomitant administration of hydroxychloroquine and low dose methylprednisolone.

Shortly after the induction of peritoneal dialysis, the patient presented with fever for the first time. All possible infectious causes were investigated including peritonitis, catheter related infection and a lupus flair, nevertheless no definite diagnosis was reached, and she was managed with empiric antibiotic treatment that led to full recovery. During the next 12 months she had 3 more hospitalizations with fever as main cause of admission and all workups were inconclusive except her last admission when high CMV viral load with 157.000 copies/mL was found. Further investigation revealed remarkably low serum immunoglobulin levels and five pulses of intravenous immunoglobulin were administered. In this direction, supplementary investigation for hypoglobulinemia was performed with repetitive measurement of immunoglobulin levels after a symptom free period which confirmed earlier findings with low IgA, IgG and IgE levels but slightly elevated IgM levels (Table 1). Then on, patient had no major complications apart from two to three episodes of upper respiratory infections and acute tonsilitis each year. Evaluating both clinical course and laboratory findings the monthly administration of intravenous immunoglobulin was initiated as acquired immunodeficiency was the most likely cause. The regimen was well tolerated.

**Table 1:** Immunoglobulin levels timeline.

	**(Normal range)**	**On CAPD initiation**	**18 months later-During CMV infection**	**Measurements while infection free (4year period)**		**2-months after IVIG initiation**	**6-months after IVIG initiation/3-months post-Tx**
IgG (mg/dl)	751–1560	406	129	<33	<33	424	1160
IgA (mg/dl)	82–453	89	54	<7	<7	<7	<7
IgM (mg/dl)	46–304	59	41	573	667	347	91
IgE (mg/dl)	5–165	16		<5	8		6

*A gradual restoration of IgG levels was achieved along with normalization of IgM levels, after induction of IVIG treatment*.

*Ig, immunoglobulin; CAPD, continuous ambulatory peritoneal dialysis; CMV, cytomegalovirus; IVIG, immunoglobulin*.

In June 2020, the patient received a cadaveric kidney transplant. The compatibility analysis revealed two shared HLA class I antigens and the donor was CMV IgG positive. Immunosuppression regimen included basiliximab 25 mg on day 0 and 4, mycophenolic acid 540 mg twice daily, tacrolimus with trough levels of 8 ng/ml directly post-operatively and methylprednisolone as well as prophylactic treatment with trimethoprim-sulfamethoxazole and valganciclovir. There was direct allograft function postoperatively and no need for renal replacement therapy. The only remarkable manifestation post-transplant was a lymphocele that was surgically managed with a peritoneal window drainage.

Treatment with IVIG was continued seamlessly and a change in immunoglobulin profile was noticed (Table 2). In more detail, IgG CMV antibodies that were absent even after the resolution of her former CMV infection were detected. Of note, our patient had also failed to acquire immunity for hepatitis B after vaccination, but HBV surface antibodies were now present. Peripheral blood immunophenotyping with flow cytometry appointed an almost absent CD20+ lymphocytes count (0.11%, 2 cells/μl).

**Table 2:** Patient's viral immunology profile changes in time.

		**On CMV infection**	**1 month later**	**6 months later**	**4-year period on infection free workups**		**4 months after IVIG initiation**	**7 months after IVIG initiation/4 months post-Tx**
CMV IgM	>22 positive	(inconclusive)	133	100	101	64.8	67	33.9
CMV IgG	>14 positive	<5		<5	<5	5	82	116
PCR CMV (copies/ml)		157.000	1.200	0		0	0	0
EBV IgM	>1 positive	<10		<1	<1	<1	<1	<1
EBV IgG	>20 positive	11		<11	<1	<1	63.36	69.18
anti-HBs (mIU/ml)	<10 negative			7.2	0.086	0.07	776.4	>1000
anti-HBc IgG	>1 positive			<1	<1	<1	<1	<1

*Major remarks are the absent CMV IgGs after the resolution of CMV viremia and the seroconversion on both CMV IgGs and anti-HBs after intravenous IVIG treatment*.

*CMV, cytomegalovirus; PCR, polymerase chain reaction; EBV, Epstein Barr virus; HBs, Hepatitis B surface antigen; HBc, Hepatitis B core antigen; IVIG, intravenous immunoglobulin; Tx, transplantation*.

## Discussion

### Kidney Transplantation, Immunosuppression and Viral Prophylaxis

Successful solid organ transplantation equals to adequate suppression of recipient's immunity response, so that both self-tolerance and a well-functioning allograft are achieved. The key target for immunosuppressive treatments in solid organ transplantation is the major histocompatibility complex (MHC) or HLA complex. When donor's HLA molecules are presented on antigen-presenting cells (APCs), they are recognized by recipient's MHC as foreign antigens giving rise to an immunity response with T-lymphocytes activation (15). T cell-mediated allorecognition and response, if not halted, is clustering with secondary signals, cytokine production and detrimental effects for the allograft, leading to rejection. Despite described efforts for self-tolerance achievement with minimization or early immunosuppression withdrawal especially in pediatric transplant patients (16), successful kidney transplantation is interconnected with a regimen that consists of two or more often three immunomodulatory agents.

Along with maintenance immunosuppression, prophylactic treatment against CMV with valganciclovir has proven efficacy especially in CMV-IgG negative patients receiving a transplant from CMV-IgG positive donor, who are regarded high risk for CMV infection and invasive disease. Treatment with valganciclovir for 3 months prevents CMV disease and ensues protection against super-infections such as herpes simplex virus while it can lead in medium and low-risk patients to lower incidence of acute rejection (17). Nevertheless, in high-risk seronegative kidney transplant recipients prophylaxis with valganciclovir is recommended for a 6 month period post-transplant (18). An alternative strategy to avoid CMV infection in transplantation is pre-emptive treatment with CMV-specific super-immune globulin, for 3 days directly postoperatively. Results from a meta-analysis in more than 5,000 solid organ transplant patients, show no superiority between valganciclovir prophylaxis and pre-emptive therapy regarding CMV disease prevention, organ rejection and superinfections. Valganciclovir was associated with more episodes of delayed CMV infections especially in seronegative recipients, while hematological toxicity was more frequent in the valganciclovir group (19). Conclusions regarding the optimal dose of valganciclovir could not be reached (19).

Close surveillance for CMV viremia using quantitative assays is an approach that allows both early detection of viral load and prompt intervention in order to avoid CMV infections. The initiation of 900 mg valganciclovir twice daily when the threshold of 400 copies/mL of CMV load is exceeded was inferior to prophylactic treatment with 450 mg of valganciclovir twice daily in CMV episodes prevention but had similar effects on rejection episodes and allograft and overall survival on the long run (20). Close monitoring of CMV replication is to be considered during the direct period post valganciclovir prophylaxis discontinuation in high-risk patients as both myelotoxicity minimization by avoiding prolonged exposure to valganciclovir and proper treatment are feasible (18, 21).

### Intravenous Immunoglobulin

IVIG preparations are produced via the extraction of immunoglobulins from healthy individuals (22). Currently IVIG is used for the management of numerous conditions which include immunodeficiency syndromes, inflammatory systemic diseases (SLE included) and hematologic conditions such as idiopathic thrombocytopenic purpura (23). A big effort has been made to describe the underlying mechanism of action for this multi-purpose therapeutic option which is characterized by both pro- and anti-inflammatory action. In brief, low dose IVIG seems to exert its pro-inflammatory action through complement and innate immunity cell activation achieved by Fc-IgG fragment binding with its receptor (FcγR) on recipient's cells giving rise to the expression of stimulatory signals and enhancing cell mediated toxicity (23, 24). While the aforementioned mechanism is thought to summarize the benefit in patients with IS, IVIG in higher doses acts as anti-inflammatory agent. Among the proposed characteristics is the scavenging of anaphylatoxins that leads to complement inhibition, the competitive action against autoantibodies and immunocomplexes regarding ligation on FcγRs decreasing granulocytes' activation, the enhancement of the inhibitory FcγRIIB expression inhibiting macrophages and modifying cytokine profile (22).

The applicability of IVIG in renal transplantation so far, involves desensitization protocols before transplantation as in ABO blood type incompatibility or in highly HLA sensitized patients, administered as monotherapy or combined with rituximab and/or plasmapheresis and finally as substitution in secondarily induced agammaglobulinemia in the context of recurrent infections (BK virus, parvovirus B19, CMV) that may cause intrinsic allograft nephropathy if left untreated (22). Although not validated with controlled studies, another indication for IVIG in kidney transplantation is antibody mediated rejection (AMR) (22) with good short-term effectiveness (25). IVIG seems to be well-tolerated and most postulated side effects concern infusion related symptoms or rare incidents of acute kidney injury due to osmotic nephrosis mainly with saccharose-containing preparations (25).

### The Case

Our patient, a young woman that reached ESKD 4 years after SLE diagnosis, remained on CAPD for 7 years. In the meantime, there were no flare-ups of her primary disease and no need for treatment step up apart from maintenance with low-dose prednisolone and hydroxychloroquine. The activity of SLE that declines in parallel with ESKD progression in time has already been described with gradual remission of lupus related manifestations and diminished serological activity (26). Nevertheless, the major concern about our patient were recurrent upper respiratory infections and the diagnosis of CMV infection. The case is further complicated by the subsequent severe hypogammaglobulinemia (Table 1) and patient's failure in acquiring immunity for CMV with undetectable CMV IgG levels (Table 2). On the other hand, there is a sustained positivity of low level CMV IgM antibodies with untraceable viral load after repeated PCR exams until present, which could be of minor importance as long as it is not accompanied by clinical and laboratory (i.e., leukopenia) manifestations or other indications of intrinsic disease.

Successful kidney transplantation is the best possible option for ESKD patients. The time dependent “burn-out” effect of autoimmunity (27), but mainly the experience gained from kidney transplantation in patients with lupus nephritis has improved outcomes and allograft survival (28). Available data regarding immunodeficiency syndromes and kidney transplantation are restricted in a small number of case reports. In such a case, a patient with primary immunodeficiency on maintenance IVIG proceeding to kidney transplantation had an uncomplicated postoperative course with direct allograft function and co-administration of immunosuppression consisting of basiliximab, tacrolimus (trough levels 3.9 ng/mL), mycophenolate mofetil and corticosteroids (29). This patient presented with allograft rejection after 2 and a half years, with de-novo donor specific antigens due to non-compliance with medication (29). In another case, a 15-year-old transplant patient treated with antithymocyte globulin, azathioprine, cyclosporine A and methylprednisolone, was diagnosed with IS 2 years post transplantation after multiple episodes of acute sinusitis and oral candidiasis, which in several instances were accompanied by acute allograft rejection episodes. However, after immunoglobulin substitution with IVIG the patient remained infection free with stable renal function (30). In a single-center case series, 3 patients with ESKD and hypo-gammaglobulinemia on IVIG treatment, received a kidney transplant with minor complications. Among them, a young woman with SLE, 6 months after transplantation suffered from extensive herpetic skin infection and oral and vaginal candidiasis that apart from antiviral and antifungal treatment was managed with minimization of her immunosuppression (31). An important aspect in this regard is the potential immunomodulatory effect of the administered IVIG and the consequent potential protection against antibody mediated rejection episodes. In this light, an additional question arises about the safe reduction of standard immunosuppressive therapy under the protective permanent IVIG administration, with the ultimate goal of reducing calcineurin inhibitor (CNI) toxicity on the one hand and preventing episodes of infection on the other.

Appropriate management of our patient's acquired immunodeficiency was initiated with the administration of IVIG 500 mg/kg every 2 weeks targeting at IgG levels > 500 mg/dl. About 4 months later a change in serologic profile was observed with positive IgG for several viruses, as shown in Table 2, and among them IgGs against surface HBV antigen. It is worth noting that the patient was a non-responder after the completion of HBV vaccination. Similar observations have already been reported in two pediatric patients (32) and in a Canadian cohort of 11 patients with ITP, 3 of whom had a seroconversion with positive anti-core IgG for HBV, post IVIG administration (32, 33). Retesting in the following weeks, after pausing IVIG revealed a return to former status, concluding that the passive transfer of pre-formed immunoglobulins was the etiology of the case (33). Overall, substitution with IVIG in solid organ transplant recipients with hypoglobulinemia seems to protect against severe infections (34) including CMV infection and lowers allograft rejection incidence (35). The restoration of IgG levels with IVIG in patients with multiple infections post-transplantation prevented this deleterious continuality (36) and improved overall survival (37).

The half-life of IVIG varies from 3 to 4 weeks, depending on the formulation administered (38). However, the question about the effectiveness and functionality of transferred immunoglobulins and whether they are indicative of immunity as reflected on the laboratory measurements still remains. The significance of this remark is further underlined when it comes to our patient's history of cytomegalovirus infection, the absence of CMV IgG antibodies even after the resolution of viremia and on the other hand the seroconversion achieved after IVIG therapy. Queries for the clinical team are still pending: the measured IgGs provide protection against CMV infection? Prophylactic treatment with valganciclovir should be prolonged after 3 or even 6 months of treatment even if there are no supporting data available (18, 21)?

## Conclusions

SLE is a disease with multiple comorbidities and ESKD may be reached not long after the diagnosis despite all efforts for tailor made immunosuppression. Even if renal replacement therapy ensures patient's survival and a good quality of life, a successful kidney transplantation remains the gold standard of treatment. Nevertheless, the management of a transplant patient can be complex when immunity dysregulations coexist, as the need for B-cell products substitution, that is, gammaglobulins via IVIG administration on one hand and T-lymphocytes suppression in order to avoid allograft rejection on the other may be simultaneously necessary. The confined data regarding kidney transplantation in patients with IS should not be discouraging as long as there is an integrated medical team and close monitoring so that early intervention according to patient's needs is undertaken.

## Data Availability Statement

The raw data supporting the conclusions of this article will be made available by the authors, without undue reservation.

## Ethics Statement

Written informed consent was obtained from the individual(s) for the publication of any potentially identifiable images or data included in this article. A written informed consent was obtained by the patient prior to writing this article.

## Author Contributions

PP wrote the first draft of the manuscript. All authors contributed to manuscript revision, read, and approved the submitted version.

## Conflict of Interest

The authors declare that the research was conducted in the absence of any commercial or financial relationships that could be construed as a potential conflict of interest.
